# The Where, When, How, and Why of Hyaluronan Binding by Immune Cells

**DOI:** 10.3389/fimmu.2015.00150

**Published:** 2015-04-14

**Authors:** Sally S. M. Lee-Sayer, Yifei Dong, Arif A. Arif, Mia Olsson, Kelly L. Brown, Pauline Johnson

**Affiliations:** ^1^Department of Microbiology and Immunology, Life Sciences Institute, University of British Columbia, Vancouver, BC, Canada; ^2^Department of Pediatrics, Child and Family Research Institute, University of British Columbia, Vancouver, BC, Canada

**Keywords:** hyaluronan, CD44, inflammation, immune cells, leukocytes

## Abstract

Hyaluronan is made and extruded from cells to form a pericellular or extracellular matrix (ECM) and is present in virtually all tissues in the body. The size and form of hyaluronan present in tissues are indicative of a healthy or inflamed tissue, and the interactions of hyaluronan with immune cells can influence their response. Thus, in order to understand how inflammation is regulated, it is necessary to understand these interactions and their consequences. Although there is a large turnover of hyaluronan in our bodies, the large molecular mass form of hyaluronan predominates in healthy tissues. Upon tissue damage and/or infection, the ECM and hyaluronan are broken down and an inflammatory response ensues. As inflammation is resolved, the ECM is restored, and high molecular mass hyaluronan predominates again. Immune cells encounter hyaluronan in the tissues and lymphoid organs and respond differently to high and low molecular mass forms. Immune cells differ in their ability to bind hyaluronan and this can vary with the cell type and their activation state. For example, peritoneal macrophages do not bind soluble hyaluronan but can be induced to bind after exposure to inflammatory stimuli. Likewise, naïve T cells, which typically express low levels of the hyaluronan receptor, CD44, do not bind hyaluronan until they undergo antigen-stimulated T cell proliferation and upregulate CD44. Despite substantial knowledge of where and when immune cells bind hyaluronan, why immune cells bind hyaluronan remains a major outstanding question. Here, we review what is currently known about the interactions of hyaluronan with immune cells in both healthy and inflamed tissues and discuss how hyaluronan binding by immune cells influences the inflammatory response.

## Introduction

The function of our immune cells is to maintain homeostasis. When immune cells detect damage or infection, they respond by making an inflammatory response that is aimed at removing the threat. The ultimate goal is to repair the damage and return the tissue to its original state. The inflammatory process is a potent, fundamental, and normally protective immune mechanism. However, if it is not properly regulated, it can result in serious damage to the host and lead to a pathological state. In fact, inflammation is thought to be at the root of many chronic conditions, from heart attacks and strokes to arthritis and type-2 diabetes. Thus, it is important to understand the factors that drive and resolve inflammation in order to better treat inflammatory diseases and identify novel therapeutic targets and new predictors of treatment efficacy.

Our skin and mucosal surfaces provide the first line of defense, and any pathogen that breaches these barriers activates innate immune cells, which trigger an inflammatory response. Macrophages are innate immune cells that reside in our tissues and play a key role in maintaining tissue homeostasis. Their primary role is to remove dead and damaged cells, and to detect and destroy invading pathogens. Dendritic cells are also innate immune cells that become activated in response to pathogens and migrate to the lymph node to activate antigen-specific adaptive immune cells (T and B lymphocytes). Once the pathogen and cell debris are removed, damaged cells and extracellular matrix (ECM) components are replaced, and tissue homeostasis is restored. One major constituent of the ECM is hyaluronan (HA), a large glycosaminoglycan under physiological conditions that becomes fragmented during infection and tissue damage, and is restored upon the resolution of inflammation. HA turnover is perturbed during inflammation and HA fragments accumulate extracellularly. These fragments are associated with propagating the inflammatory response, whereas full-length high molecular mass HA is associated with the resolution of inflammation. While all immune cells express the HA receptor, CD44, not many bind HA under homeostatic conditions. However, this changes when immune cells become activated. In this review, we discuss what is known about the interactions between immune cells and HA during homeostasis and inflammation.

## HA Turnover during Homeostasis and Inflammation

**Figure 1:**
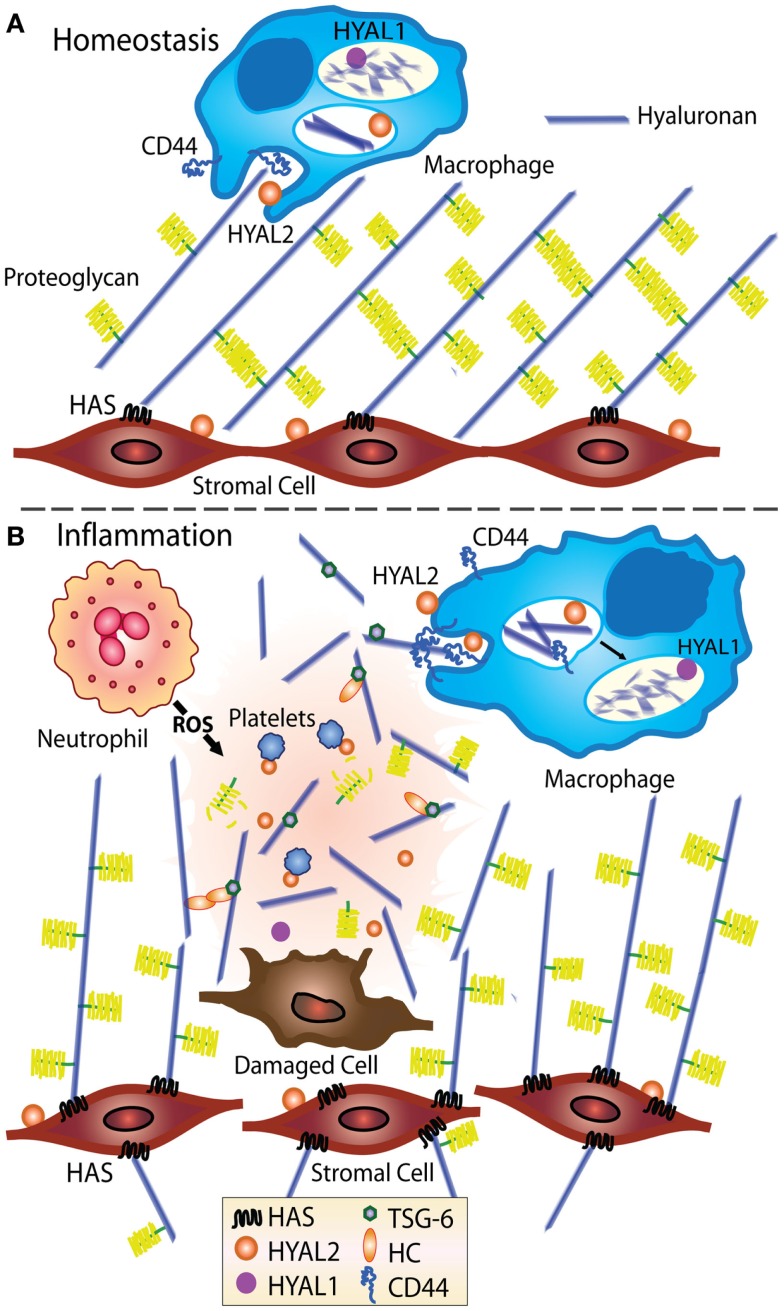
**Model showing HA turnover at homeostasis and during inflammation**. At homeostasis **(A)**, stromal cells produce ECM, including HA, which becomes decorated with proteoglycans such as versican (14–16), indicated by the yellow brush structures. HA is turned over in tissues, likely by CD44-mediated cellular uptake by fibroblasts and macrophages (17), then degraded by Hyal 1 and 2 (12, 13). **(B)** During inflammation, the levels of HA increase and the ECM becomes susceptible to damage and fragmentation (18–20). Inflammation induced HA binding proteins such as TSG-6 bind and crosslink itself or the heavy chain of IαI (HC) to HA (21, 22) and in some situations such as chronic inflammation, HA deposits may develop (3, 18). CD44-mediated uptake of HA fragments by macrophages is thought to play an important role in resolving inflammation (19).

HA is widely distributed throughout all the tissues in the body with up to 50% being present in the skin. HA is found at high levels in the umbilical cord (~4 mg/ml) and synovial fluid (~2 mg/ml); it is prevalent in the vitreous of the eye (~100–400 μg/g of wet tissues) and the dermis of the skin (~500 μg/g wet tissue); and present at 10–100 ng/ml in the blood (1, 2). It is hygroscopic in nature and has viscoelastic properties making it a useful lubricant in joints. HA comprises repeating units of d-*N*-acetyl glucosamine and d-glucuronic acid. HA is often confined to specific areas within tissues, for example, HA is present around blood vessels and bronchioles in the lung (3). At homeostasis, HA production is balanced by its cellular uptake and degradation (4). Cellular HA synthases (HAS 1–3) and hyaluronidases (Hyal 1–3) mediate the turnover of HA [reviewed in more detail elsewhere (5–11)]. HA catabolism can occur locally by nearby cells involving CD44-mediated uptake, partial degradation by Hyal 2, and further degradation in the lysosome by Hyal 1 [(12, 13); see Figure 1]. Alternatively, HA can drain into the lymphatics and be degraded at a distant site such as the liver (2).

During inflammation, an increase in HA is accompanied by a decrease in chain length, possibly due to altered HAS and Hyal activities (3, 18) or to cleavage by reactive oxygen and nitrogen species produced by activated immune cells (23, 24). Macrophages are thought to be involved in HA uptake and the removal of HA fragments (19), and stromal cells are a major source of newly synthesized HA, see Figure 1. Upon the resolution of inflammation, HA production and cellular turnover return to normal and the high molecular mass form predominates again.

## The Different Forms of HA

### At homeostasis

#### HA as part of the extracellular matrix

Hyaluronan is secreted from the cell and forms pericellular or extracellular matrices presumably after cleavage and release from the cell surface. HA is a major component of the ECM and at homeostasis, extracellular HA is found predominantly in its high molecular mass form of over 1000 kDa (10, 25, 26). HA chains occupy a large hydrodynamic volume in solution (27) and can associate with collagen in extracellular matrices. Proteoglycans such as versican and aggrecan bind to HA and this could create a stable network under homeostatic conditions (14–16).

### During inflammation

#### HA fragments

Both damage and inflammatory conditions can cause the fragmentation of HA, which is considered fragmented when its molecular mass falls below 500 kDa. Studies in the lung tissue have reported the detection of 500 kDa HA fragments after bleomycin-induced inflammation (19), 70 kDa fragments after cigarette smoke-induced chronic obstructive pulmonary disease (COPD) (18), and 100–200 kDa fragments in the bronchial alveolar lavage fluid (BALF) after ozone-induced airway hyper-responsiveness (20). There is evidence that HA fragmentation can result from degradation by reactive oxygen species (ROS) that are produced by neutrophils (23, 24), or by enzymatic cleavage by Hyal 1 or 2 that perhaps have escaped from dying cells. Hyal 2 and Hyal 1 both work optimally at acidic pH and break down HA to 20 kDa and small oligomers, respectively (28). Alternatively, an increase in HA fragments may be the result of HA synthases making smaller chains of HA (3, 18).

#### HA oligomers

Under normal conditions, small oligomers of HA should only be present in the lysosome as HA is degraded into tetrasaccharides by Hyal 1. Hyal 1 has been reported in the serum, but neither the source nor its activity is known (28). Thus, one can predict that the smallest oligomers of HA would only be present under severe inflammatory conditions when there is significant cell death and release of the lysosomal contents. It is these smaller oligomers that are thought to activate dendritic cells (29, 30). As CD44 binds to these oligomers with very low avidity, they may activate dendritic cells by interacting with other receptors such as TLR2 or 4, although this remains to be shown directly.

#### HA complexes

*In vitro*, pericellular cable-like structures of HA can be induced in cells in response to inflammatory stimuli such as polyI:C, a viral RNA mimic (31, 32), or to tunicamycin, an ER stress and N-glycosylation inhibitor (33). These HA cables are also positive for the inter-alpha-trypsin inhibitor (IαI) (32). The heavy chains (HC or serum HA-associated protein, SHAP) of IαI can become covalently bound to HA and this is thought to crosslink and stabilize HA (34). The human monocytic cell line U937 and human peripheral blood mononuclear leukocytes, which do not bind soluble HA, can bind to these cables (31, 32, 35), although only at 4°C as at 37°C the HA is taken up by the U937 cells. (36). HA cables have been induced in a variety of cell types *in vitro*, including smooth muscle cells from the lung (37) and colon (32), in airway (38) and renal epithelial cells (39), as well as epidermal keratinocytes (40). However, HA cables have yet to be described *in vivo*, although HA and IαI co-staining has been reported in a human case of inflammatory bowel disease (32).

TNFα-stimulated gene-6 protein (TSG-6) is a multifunctional protein that has a HA binding module and binds HA under acidic conditions (41). TSG-6 production is greatly increased with inflammation and is associated with tissue remodeling (41). TSG-6 is the enzyme that catalyzes the covalent addition of the HC from IαI to HA (21, 22, 42), which can lead to the crosslinking and aggregation of HA (34). Interestingly, TSG-6 and the attachment of the HC from IαI enhance the binding of HA to CD44 (43, 44). Furthermore, these HA–HC complexes are found as HA deposits *in vivo* during persistent inflammation in the lung and TSG-6 has been shown to promote these deposits (3, 45). However, the function of these HA–HC complexes in inflammation and tissue remodeling is still being explored.

## HA Binding by Immune Cells at Homeostasis

### HA binding by alveolar macrophages

Under homeostatic conditions, without infection or inflammation, the majority of developing and mature immune cells do not bind HA, as assessed by flow cytometry using fluoresceinated HA (Fl-HA, see Box 1). In fact, alveolar macrophages are the only immune cells that have been shown to bind high levels of HA under homeostatic, non-inflammatory conditions, in both rodents and humans [(46–48); see Table 1]. Alveolar macrophages reside in the respiratory tract and alveolar space, between the epithelial layer and surfactant, where they are responsible for the uptake and clearance of pathogens and debris. In the absence of these macrophages, the immune response is exacerbated (49), indicating that these scavenger cells also have a role in limiting inflammation, perhaps by clearing debris and removing inflammatory stimuli. Alveolar macrophages take up HA in a CD44-dependent manner, which is then delivered to the lysosomes and subsequently degraded (17). HA is present in the connective tissue space during lung development, but is reduced as the number of CD44-positive macrophages increases (50). Fetal alveolar type II pneumocytes produce HA (51), which is thought to associate with the pulmonary surfactant. However, in adults, it is less clear if mature pneumocytes make HA and most of the HA in the lung tissue is found lining blood vessels and bronchioles (3, 50). There seems to be two possible explanations why alveolar macrophages constitutively bind HA: (1) to bind to the HA producing pneumocytes to help anchor themselves in the alveolar space or (2) to internalize HA or HA fragments and help keep the alveolar space free of debris.

Box 1. Evaluation of HA binding by flow cytometry. Hyaluronan from rooster comb (1000–1500 kDa) or commercially available HA of specific molecular mass is conjugated to fluorescent dyes, using the method of de Belder (52), or indirectly using a coupling reagent. Fluoresceinated HA (Fl-HA) used in flow cytometry provides a useful means to evaluate surface HA binding, HA uptake, and CD44-specific HA binding using HA-blocking CD44 mAbs such as KM81 or KM201 (53). To date, all experiments indicate that the HA binding on immune cells is mediated by CD44 [(54, 55), and reviewed in Ref. (56, 57)].

High molecular mass HA (>1000 kDa) binds to CD44 with a higher avidity than medium (~200 kDa) or low (<20 kDa) molecular mass HA fragments, and thus high molecular mass Fl-HA is routinely used to evaluate HA binding by immune cells. CD44 can bind monovalently to 6–18 sugars of HA, with a noticeable increase in avidity when the HA reaches 20–38 sugars in length, suggesting that divalent binding is occurring (58). The avidity will increase with increasing length as more CD44 molecules are engaged. Ultimately, the strength of Fl-HA binding depends on the size of HA as well as the amount, density, and type of CD44 at the cell surface. Flow cytometry allows us to determine relative HA binding abilities as it can distinguish cells that bind different amounts of Fl-HA. The pretreatment of cells with hyaluronidase (which is then washed away) can determine if CD44 is binding to endogenous HA and thus blocking the binding of Fl-HA. It is thus a useful technique to gain insights into the HA binding abilities of CD44. Commercial sources now provide specific molecular sizes of purified soluble HA that are low in contaminants and endotoxin. However, it is important to keep in mind that purified soluble chains of HA may not always be the form encountered *in vivo*.

**Table 1:** **HA binding ability of immune cells**.

Cell type	Stimulation	Type of HA binding	HA receptor	Reference
Monocyte (human)	TNFα, LPS, IL-1, IFN-γ	Inducible	CD44	(59–61)
Alveolar macrophages (human, rodents)	None	Constitutive	CD44	(17, 46 –48, 50)
Peritoneal macrophage (mouse)	LPS with IFNγ, or IL-4	Inducible	CD44	(62)
Bone marrow-derived macrophages (mouse)	LPS with IFNγ, TNFα, or IL-4	Inducible	CD44	(62)
Monocyte-derived DC (human)	CD40L expressing fibroblasts	Inducible	CD44	(63)
B cells (human, mouse)	PMA, IL-5, LPS	Inducible, a subset binds	CD44	(64 –67)
T cells (mouse)	PMA/ionomycin, CD3 antibodies, specific antigen, or superantigen	Inducible, often a subset binds	CD44	(54, 55, 68)
CD4+ CD25+ T regulatory cells (human and mouse)	CD3 +/− CD28 activation	Inducible, a subset binds	CD44	(69, 70)
Neutrophil (mouse)	LPS induced liver inflammation *in vivo*	Binding to SHAP modified HA	CD44, not RHAMM	(71)
NK cells (mouse)	IL-2, IL-15	Inducible, a subset binds	CD44	(72)
Platelets (mouse)	None	Constitutive	CD44	(73)

### The HA binding status of dendritic cells

There is very little direct evidence that immature or mature dendritic cells bind HA, HA fragments, or oligomers of HA, despite reports that HA fragments and small oligomers of HA stimulate dendritic cells to produce proinflammatory cytokines (29, 30, 74). Dendritic cells have been reported to express HA and HA-synthesizing and degrading enzymes (75), with human monocyte-derived dendritic cells expressing primarily HAS 3 (76). However, it is not clear whether CD44 on either immature or activated dendritic cells is capable of interacting with HA. Unlike macrophages, which stay in the tissues to fight infection and maintain homeostasis, activated dendritic cells migrate to the draining lymph node, where they present antigen to naïve T cells. A HA binding peptide, Pep-1, reduced dendritic cell clusters and antigen-induced T cell proliferation, however, Pep-1 acted on the T cells, suggesting that T cells make HA (75). Non-endotoxin tested HA upregulated costimulatory molecule expression on bone marrow-derived dendritic cells, which facilitated T cell proliferation, while CD44 on the T cells promoted clustering with the dendritic cells (74). However, since endotoxin is a common contaminant in some HA preparations and can have similar effects on dendritic cells, additional steps are needed to exclude an endotoxin effect. Supernatants from Th1 clones also promoted HA-dependent adhesion between human monocyte-derived dendritic cells and activated T cells or Th1 or Th2 clones, providing evidence for a dendritic cell-HA:CD44-T cell interaction (76). However, as we discuss below, naïve T cells express low levels of CD44 and have a low avidity for HA, making this mechanism unlikely to play a key role in the initial contact and activation of naïve T cells, unless dendritic cells produce a form of HA that enables naive T cells to bind. It could, however, play a role in activating memory T cells, which express higher levels of CD44 and have a greater propensity to bind HA (54).

### The lack of HA binding by naïve T cells

Activated dendritic cells present antigen to naive T cells, which stimulates their activation, proliferation, and differentiation into effector T cells. Early studies showed that CD44 monoclonal antibodies (mAbs) could provide a costimulatory signal that together with a signal from the T cell receptor (TCR) activates T cells (77–79). As CD44 is present in lipid rafts along with the tyrosine kinase Lck (80), its crosslinking was thought to help Lck activation and bring it into contact with the TCR signaling complex (81). Although these antibodies crosslink CD44 and enhance TCR signaling, there is limited evidence that HA can do this, perhaps because naïve T cells have a very low avidity for HA. Data from naïve T cells isolated from C57Bl/6 mice show that naïve T cells express low levels of CD44 and do not bind Fl-HA (54, 55, 68). However, one study showed that immobilized HA could augment PMA or CD3-induced proliferation of human peripheral blood T lymphocytes and could augment IL-2 production from CD3-stimulated CD4 T cell clones (82). In this study, HA binding did not become apparent until 1–2 days after stimulation.

## HA Binding by Immune Cells during Inflammation

Activation of immune cells by proinflammatory cytokines, inflammatory stimuli, and by antigen recognition can all induce HA binding by CD44 [reviewed in Ref. (56); see Table 1]. HA binding in response to these stimuli is generally accompanied by an increase in CD44 expression and typically takes 2–3 days to reach maximal HA binding levels. Flow cytometry can indicate a shift in HA binding or identify a specific subset of binding cells (see Box 1 for more details). Table 1 shows the stimuli that induce HA binding in the various immune cells. Although neutrophils are major inflammatory phagocytic cells, these cells do not bind Fl-HA and are normally recruited to inflammatory sites independently of CD44 (83). However, as we discuss later, HC-modified HA provides a means of neutrophil recruitment to inflamed liver (71). Below, we will focus on the HA binding abilities of monocytes, macrophages, dendritic cells, and T cells during inflammation.

### The induction of HA binding by monocytes and macrophages

Human monocytes in the blood bind negligible amounts of soluble Fl-HA (59–61), but are induced to bind over a period of 2–3 days when activated *in vitro* by inflammatory cytokines such as TNFα and IL-1β, and the inflammatory agent, LPS (see Table 1). Similarly, M-CSF-induced mouse bone marrow-derived macrophages and *ex vivo* peritoneal macrophages do not bind Fl-HA until induced by proinflammatory agents (62). Treatment of TNFα-induced human monocytes with IL-4 prevents the induction of HA binding (60), whereas IL-4 treatment alone induced HA binding on mouse bone marrow-derived macrophages (62). Both the transcription and post-translational modifications of CD44, such as glycosaminoglycan or carbohydrate addition, can influence HA binding. Decreases in sialylation and changes in the chondroitin sulfate modification to CD44 can modulate HA binding by human monocytes (84) and mouse macrophages, respectively (62). Macrophages are found in many locations in the body, with resident populations in the spleen, liver, skin, gut, lung and the alveolar space, brain, and the peritoneum, but only the alveolar macrophages bind substantial amounts of HA under homeostatic conditions. This suggests that the environment of the macrophage influences its ability to interact with HA, and raises the possibility that these macrophages may be induced to bind HA when the environment or cytokine milieu changes upon infection or inflammation.

Why inflammation induces HA binding on monocytes and macrophages is not well understood. Possible explanations include: (1) activated monocytes and macrophages use HA as a substrate to aid in migration toward the site of infection; (2) an enhanced ability to bind HA or HA–HC complexes to retain activated immune cells in the tissue at sites of inflammation; (3) an enhanced ability to bind, take up, and degrade HA, HA fragments, or HA–HC complexes via CD44, thereby helping reduce inflammation and promote tissue repair; (4) HA binding provides a supportive environment for these cells, either by providing a direct survival signal to the cell or by creating a cytokine-rich environment that aids in their survival, proliferation, or function.

### HA binding by activated T cells

**Figure 2:**
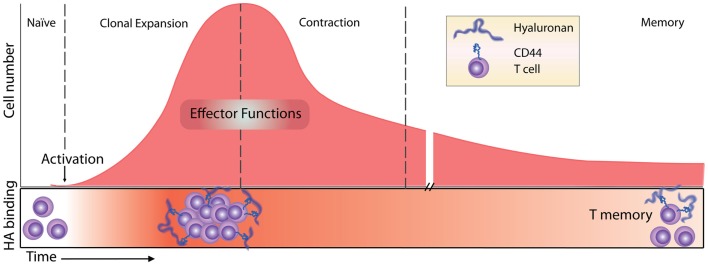
**Timeline of the T cell response and HA binding**. Before immune challenge, naïve T cells express low levels of CD44 and have low affinity/avidity for HA (55). Following activation, HA binding is induced (55, 68) and marks the most proliferative, activated T cells (54). HA binding on effector T cells has a role in T cell extravasation into inflamed peritoneum (68), and marks the majority of cytotoxic CD8 T cells (55). Contraction and generation of memory CD8 T cells result in memory cells that maintain high levels of expression of CD44 but only a percentage of them (approximately 30%) bind HA (54).

In contrast to unstimulated naïve T cells, antigen-induced activation of T cells induces HA binding, coincident with an increase in CD44 expression, and this is now well-established both *in vitro* and *in vivo* [(54, 55, 68); see Figure 2]. *In vitro*, the induction of HA binding is transient, peaking at 2–3 days, whereas it is more sustained *in vivo*, reaching its maximum around 5–8 days (54, 55). The absence of any significant Fl-HA binding on naïve T cells until 2–3 days, after at least the first division, also argues against a model where HA on dendritic cells facilitates CD44-mediated naïve T cell adhesion and the initiation of T cell activation. As T cell activation and proliferation proceeds, HA binding increases, as does the ability of T cells to roll under flow on a HA substrate (85) and the ability of superantigen-activated T cells to extravasate to the inflamed peritoneum in an HA-dependent manner (86). Thus, one function of the upregulation of HA binding by activated T cells may be to guide the effector T cell to the site of infection. This later induction of HA binding after activation and some proliferation suggests a role beyond the initial contact and activation step. Notably, the strength of the signal received by the T cell dictates the level and percentage of T cells that are induced to bind HA, with cells binding the most HA being the most proliferative (54). Despite these correlations, no evidence was found to support HA-stimulated proliferation. However, in another study focusing on CD4^+^ CD25^+^ T cells, HA was found to stimulate IL-2 production and sustain FoxP3 expression (87). 4-methylumbelliferone, a compound that can inhibit HA synthesis (88, and also see article in this research topic), prevented T cell proliferation and IL-2 production, suggesting that HA synthesis by the T cell itself is important for this effect (89). Thus, there is some evidence that HA production and/or the binding of high molecular mass HA on activated T cells may enhance IL-2 production, which may either prolong T cell proliferation or, if acting on regulatory T cells (Tregs), may limit T cell activation. The outcome of an interaction of activated T cells with high molecular mass HA may also depend on the differentiation state of the T cell, as re-activated T cells exposed to high molecular mass HA undergo a rapid form of activation-induced cell death, as observed in human Jurkat T cells and a subpopulation of splenic T cells from mice (90).

### HA binding by effector CD4 and CD8 T cells

After naïve T cell activation and proliferation, CD4 T cells differentiate into various effector T cells (Th1, Th2, Th17, Tregs), and CD8 T cells become cytotoxic effector cells. Both CD4 and CD8 effector cells leave the lymph node and migrate to the infected tissue to fight the infection. After the initial induction of HA binding during the proliferative phase, it is less clear how long these cells retain their HA binding abilities. *In vitro* evidence indicates that after antigen-induced HA binding in 50% of ovalbumin- (Ova-) specific OT-I CD8 T cells 2 days post activation, only 6% retain HA binding at day 6 (54). *In vivo*, antigen-induced HA binding peaks 5 days post infection, marking approximately 50% of the OT-I CD8 T cells, and this drops to 14% by day 10, indicating that HA binding is more sustained *in vivo* although it also declines after the peak of the proliferative phase (54). In other mouse studies, the percent of HA binding cells peaked at day 7–8 and the HA-positive population contained the majority of the cytotoxic CD8 T effector cells (55), consistent with the HA-negative cells being naïve cells. However, since both soluble HA and HA-blocking CD44 mAbs had no effect on allogeneic killing, HA binding was not implicated in the killing function of these cells (55). After the peak of the response, many effector T cells undergo apoptosis during the contraction phase and only long-lived memory cells remain. Following *Listeria*-Ova infection and the development of T memory cells *in vivo*, about 30% of the Ova-specific OT-I CD8 memory T cells in the bone marrow and spleen bind Fl-HA 30 days after infection [(54); see Figure 2].

CD4 T cells differentiated *in vitro* to become either Th1 or Th2 bind slightly higher levels of Fl-HA than naïve T cells, with Th2 cells binding slightly more than Th1, but the binding is still at a very low level (91, 92). Nevertheless, this low-level binding is sufficient to allow them to roll and adhere on TNFα-inflamed endothelium *in vivo* (91). In another study, CD44 promoted T effector cell survival from Fas-mediated contraction, and was required for the formation of Th1 memory cells, but the effect of HA was not examined (93). Thus HA binding by effector T cells may assist in their recruitment to inflammatory sites and the interaction of HA with CD44 in the tissues may provide a survival signal for the effector T cell.

### HA binding by activated CD4 T regulatory cells

Tregs express CD4 and CD25, as do activated CD4 T cells, and so are further characterized by expression of the transcription factor, FoxP3 (94). Firan and colleagues activated CD4^+^CD25^+^ T cells isolated from BalbC mice, separated them into HA binding and non-binding populations, and found that the HA binding fraction was functionally more suppressive (69). The activation of human peripheral blood CD4^+^CD25^+^ T cells induced a subpopulation to bind HA and this correlated with the highest expression of CD44 and FoxP3 (70). The addition of 20 μg/ml of high molecular mass HA (1.5 × 10^6^ Da) maintained FoxP3 expression under limiting levels of IL-2, and enhanced their suppressive ability *in vitro*. At a higher concentration (100 μg/ml), HA directly reduced CD4 T cell proliferation (70). Both high molecular mass HA and the crosslinking of CD44 provided a costimulatory signal that augmented IL-2, FoxP3 expression, and IL-10 production in the human CD4^+^ CD25^+^ T cell population (87). Furthermore, HA or CD44 crosslinking activated p38 and ERK1/2-dependent pathways that induced IL-10 producing regulatory T cells (TR1) from FoxP3-negative cells (95). Together, this suggests a role for high molecular mass HA in limiting T cell proliferation either indirectly via supporting Tregs or directly when given in higher amounts to CD4 T cells.

It is clear from the above sections that HA binding by T cells is not restricted to activated Tregs, suggesting a more general function for HA binding in activated CD4 and CD8 T cells. Since HA binding labels the most proliferative, functionally active T cells, HA may exert its effect by aiding in the production of IL-2 under limiting conditions. Alternatively, perhaps HA localizes activated T cells to a specific area in the lymph node, where they have optimal access to cytokines and growth factors, or maybe HA binding itself provides a survival signal for activated T cells.

## The Function of HA Binding by Immune Cells

Previous reviews have discussed HA binding by immune cells (56, 85), and have detailed what is known regarding the role of HA (96) and CD44 in inflammation and inflammatory diseases (57, 97, 98). Many diseases involve an inflammatory component and it has become apparent that HA levels are increased in many tissues upon inflammation. Here, we describe the effects of HA interactions with immune cells during inflammation with a particular focus on lung inflammation.

### The role of CD44 and HA in leukocyte recruitment to inflammatory sites

#### Upregulation of HA on microvascular endothelium facilitates T cell recruitment

At the first signs of damage or infection in a tissue, ensuing danger signals are received by macrophages that induce the secretion of inflammatory stimuli such as TNFα and IL-1β, which act on the endothelium in the microvasculature. This leads to the upregulation of adhesion molecules that facilitate leukocyte recruitment to the inflamed tissue. HA is one adhesion molecule that is upregulated on microvascular endothelial cell lines (99). Under flow conditions, T cells can roll on HA via CD44 both *in vitro* (100, 101) and *in vivo* (91), implying that the upregulation of HA on activated endothelial cells will facilitate T cell recruitment to inflamed tissues. Indeed, a CD44-mediated interaction with HA is important for T cell extravasation into the peritoneum in a model of superantigen-driven T cell activation and inflammation (86). However, given the well-established roles of the selectin molecules in leukocyte recruitment (102), the CD44–HA interaction may provide an additional and possibly redundant mechanism to facilitate T cell extravasation. The fact that the majority of CD44-deficient leukocytes still reach inflammatory sites (57) supports this idea.

#### HC-modified HA facilitates CD44-mediated neutrophil recruitment to liver sinusoids

CD44- and HA-dependent rolling on endothelium is not a factor for neutrophil recruitment to inflammatory sites (83). However, CD44-mediated adhesion to HA is a key factor in neutrophil adhesion to inflamed liver sinusoids in endotoxemic mice (71). Leukocyte recruitment to liver sinusoids proceeds in the absence of rolling and involves both integrin- and CD44-dependent mechanisms (103). LPS recognition by liver endothelial cells induces the deposition of HC (SHAP) on HA that is constitutively expressed by the liver sinusoids, and this leads to CD44-dependent adhesion of neutrophils to HA (71, 104, and also see article in this research topic).

#### CD44-mediated HA binding facilitates eosinophil and Th2 cell recruitment to the allergic lung

In experimental pulmonary eosinophilia that is induced by administration of *Ascaris suum* extract, HA-blocking CD44 antibodies prevented lymphocyte and eosinophil recruitment into the BALF (105). A follow-up study using CD44-deficient mice and the house dust mite allergen concluded that the loss of CD44 affected Th2, but not Th1 recruitment to the BALF (92). This suggests that HA and CD44 play a role in the recruitment of eosinophils and Th2 cells in an allergic response in the lung.

### A role for HA fragments and deposits in promoting inflammation

#### The proinflammatory effects of HA fragments on immune cells

##### In vitro

**Figure 3:**
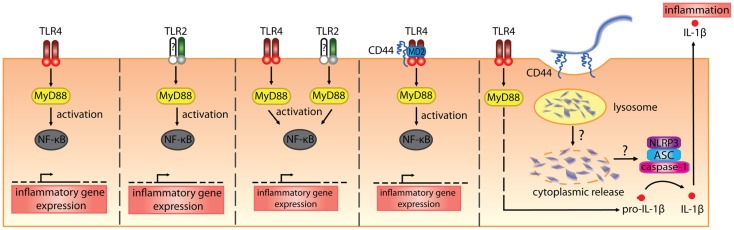
**The various receptors implicated in mediating the proinflammatory signals induced by HA fragments**. From the left, HA fragments ranging from 500 kDa and below have been reported to stimulate proinflammatory cytokine production via TLR4 alone, TLR2 alone, both TLR2 and TLR4, a complex of TLR4 involving CD44, or via TLR4- and CD44-mediated uptake that leads to inflammasome activation (110–114). In the first three panels, the TLRs signal via MyD88 to activate NF-κB and induce proinflammatory cytokine gene expression. In the fourth panel, CD44, together with TLR4 and MD-2, is needed for HA-stimulated proinflammatory cytokine production. In the fifth panel, TLR4 signals via MyD88 to produce pro-IL-1β and the uptake of HA via CD44 leads to the breakdown of HA which, through some unknown mechanism, leads to HA oligomers in the cytoplasm and these trigger NLRP3 inflammasome activation. This leads to cleavage of pro-IL-1β and the generation of IL-1β (114).

Hyaluronan fragments were first reported to stimulate chemokine and proinflammatory cytokine expression and NFkB-mediated iNOS expression in macrophage cell lines in the 1990s (106–109). At first, it was thought that CD44 mediated the effect of these HA fragments, which were quite large (470, 200–280 kDa), but later studies began to implicate the toll-like receptors, TLR2 and/or TLR4 (Table 2; Figure 3). While CD44 can bind HA fragments on activated cells, it is not clear if this alone leads to proinflammatory cytokine secretion. Instead, TLR4 (110), TLR2 (111), TLR2 and TLR4 (112), a complex of CD44 and TLR4 (113), or a combination of TLR4, CD44 and activation of the NLRP3-mediated inflammasome (114) have all been implicated in mediating HA fragment-induced proinflammatory signals (see Figure 3). TLR4 was required for HA oligomers to initiate dendritic cell maturation and proinflammatory cytokine production from both human and mouse *in vitro*-derived dendritic cells (29, 30) and 200 kDa HA fragments stimulated dendritic cell maturation in a CD44-independent manner (74). However, whether HA fragments can directly activate TLRs remains uncertain, as contaminants such as LPS can produce similar results and the direct binding of HA to TLRs has not yet been demonstrated. This needs to be determined to establish HA as a *bone fide* TLR ligand.

**Table 2:** **The effect of HA and HA fragments on macrophages and dendritic cells**.

Cell type	HA size (kDa)	HA source	Effect	Molecule	Steps to exclude LPS	Reference
**MACROPHAGES AND MONOCYTES**						
BMDM	40–80	Bovine trachea	Induced IL-1β, TNFα mRNA	CD44	Used LPS hyporesponsive mouse	(106)

BMDM and MH-S cell line	6000	Rooster comb	No effect	–	Not specified	(107)
	474, 267	Sonicated	Activated NFκβ	

MH-S cell line and human inflammatory alveolar Mϕ	6000	Rooster comb	No effect	CD44	Endotoxin tested, polymyxin added	(109)
	470	Sonicated	Induced MIP1α, MIP1β, IP-10, RANTES, MCP-1 mRNA in mouse and IL-8 in human	
	280	Human umbilical cord	
	35	
	6 mer	Rooster comb	
	2 mer	Not specified	No effect	

MH-S cell line, BMDM	200	Human umbilical cord	Induced iNOS mRNA		Used LPS hyporesponsive mouse, polymyxin added	(108)
			Activated NFκβ	

Elicited peritoneal Mϕ	280	Human umbilical cord	MIP1α, MIP1β, RANTES, IL-12 mRNA		Used LPS hyporesponsive mouse, endotoxin present, polymyxin added	(115)

Peritoneal Mϕ	135	Digested from bacterial HA	Induced MIP-2 and TNFα	TLR2	CD44, TLR2/4 KO mice. Endotoxin tested (<40 pg/mg HA), Hyal digestion, polymyxin added	(112)
				TLR4	
	200	Patient serum^a^	Induced MIP-2, MIP1α, KC	Not CD44	

Elicited peritoneal Mϕ	6000	Rooster comb	No effect	–	Used LPS hyporesponsive mouse, endotoxin tested solutions, polymyxin added	(111)
	200	Human umbilical cord	Induced MIP1α, MIP1β, KC, MCP-1, RANTES, TNFα mRNA	TLR2	

MH-S cell line and human THP-1 cell line	Not specified	Human umbilical cord	Induced MIP-2, TNF α, GM-csf, RANTES IL-1α, and *Mmp13, Tgfb2* mRNA, and IL-8 (human)	CD44	Endotoxin removed, DNA free	(113)
				TLR4	

MH-S cell line and elicited peritoneal Mϕ	200	Human umbilical cord	Induced TNFα and KC secretion	–	Used LPS hyporesponsive mouse	(116)

MH-S cell line and elicited peritoneal Mϕ	500–730	Rooster comb	No effect	CD44	Used a LPS hyporesponsive mouse, Hyal digestion, endotoxin removal	(114)
	Up to 500	Human umbilical cord	Induced MIP-2 and pro-IL-1β via TLR4. Induced IL-1β via CD44, Hyals and NLRP3	TLR4	
				NLRP3	
	4–18 mers	Not specified	Induced MIP-2		

Raw264 and MH-S cell line	Specific sizes 11–970	Acid hydrolysis of bacterial HA	No effect on NO or TNFα	–	Endotoxin tested <0.01 EU/ml	(117)
**DENDRITIC CELLS**						
BMDC, human blood-derived DCs	1000	Rooster comb, sonicated	No effect	–	Endotoxin tested (<0.06 EU/ml or <0.1 ng/ml LPS), polymyxin added	(29, 30)
	80–200	
	4–14 mers	Hyal digestion of sonicated HA	Induced HLR-DR expression and IL-1β, TNFα, IL-12. Activated NFκβ. Activated MAPK/p38 and NFκβ	TLR4 not CD44 or RHAMM	

BMDC	>570	Not specified	Upregulated CD40, CD80, CD86	Not CD44	Polymyxin added	(74)

BMDC	200	Human umbilical cord	Induced MIP1α		Used LPS hyporesponsive mouse, polymyxin added	(111)

BMDC	2–12 mers	Commercial source	Unable to activate DCs *in vitro*	–	–	(118)

*All cells are murine unless otherwise stated*.

*BMDM and BMDC, bone marrow-derived macrophages and dendritic cells, respectively; Mϕ, macrophages; Hyal, hyaluronidase. MIP-2 and KC are also known as CXCL2 and CXCL1, respectively*.

*^a^Patient serum was from patients with acute lung injury. Items in blue indicate no proinflammatory effect was observed with HA*.

In Table 2, we summarize the reported proinflammatory effects of HA and HA fragments on macrophages and dendritic cells *in vitro*. Proinflammatory effects have been reported with a wide range of HA fragment sizes: from HA oligomers [2–18 mers] to HA fragments ranging from 5 to 500 kDa. Interestingly, we note that HA fragments derived from rooster comb did not generally elicit proinflammatory responses whereas human umbilical cord-derived HA did. Human umbilical cord HA is not as pure as HA purified from rooster comb (some preparations are FDA approved for injection into humans), leaving open the possibility that the effects seen with human umbilical cord HA maybe due to LPS contamination. Indeed, in our hands HA from human umbilical cord but not rooster comb, tested positive for endotoxin (unpublished data). Although endotoxin levels were checked and polymyxin B was added to bind to LPS in some cases, possible residual contamination with TLR agonists remains a concern. In some cases, DNA contamination of HA was responsible for the proinflammatory activity on monocytes (119). Alternatively, there could be something different about the HA isolated from human umbilical cord as it does appear to be of a lower average molecular mass compared to rooster comb HA. Specific sizes of HA are now available from commercial sources that are purified from bacteria and certified endotoxin-free, and so it will be of interest to see if similar data are obtained with these HA preparations. Indeed, studies monitoring endotoxin levels more closely are now emerging. One report on glomerular mesangial cells shows that hyaluronidase is contaminated with LPS and suggests that HA provides a protective barrier for cells, which when cleaved exposes the TLRs (120). Others report no proinflammatory effect of endotoxin-free HA oligomers (118) or HA fragments on immune cells [(117); see Table 2]. Thus, further work with endotoxin-free HA is needed to substantiate whether HA fragments induce proinflammatory cytokine production and whether they do this by directly engaging TLRs.

##### In vivo

Ozone-induced airway hypersensitivity is associated with increased hyaluronan in the BALF, and CD44- and IαI-deficient mice were protected from this airway hypersensitivity implying a role for CD44 and IαI in driving the hypersensitivity (20). Interestingly, the instillation of fragmented, but not full-length HA, partially mimicked airway hyper-responsiveness (20). Ozone- and fragmented HA-induced airway hyper-responsiveness were also reduced in TLR4- and MyD88-deficient mice, leading to the conclusion that fragmented HA responses require TLR4 *in vivo* (121).

Using a mouse model of allergic contact dermatitis, Martin and colleagues found that inhibition of ROS and HA breakdown prevented sensitization and contact hypersensitivity (118). ROS stimulated hyaluronidase activity, which rapidly degraded HA in the epidermis, in a similar mechanism to that described in bronchial epithelial cells (122). Although this *in vivo* hypersensitivity reaction requires TLR2 and TLR4 (123), the authors have been unable to activate dendritic cells *in vitro* using commercially available HA oligomers of 2–12 sugars in length (118). These studies show that HA fragmentation occurs *in vivo* and is required for hypersensitivity responses, as are TLRs. If HA fragments do not act directly via the TLRs, perhaps TLRs are activated indirectly, by something that is released or produced upon their fragmentation. The involvement of IαI suggests that *in vivo* HA fragments may arise from HA–HC deposits and thus may contain other proteins and proteoglycans besides HA. This also raises the cautionary note that HA fragments and HA–HC complexes generated *in vivo* may be quite different from the purified forms of HA used *in vitro* studies and in Fl-HA labeling. Further work is thus needed to understand the HA-driven inflammatory mechanisms observed *in vitro* and *in vivo*.

#### HA deposits promote an allergic inflammatory response in the lung

In a mouse model of allergic asthma using Ova, a significant increase in HA deposition is observed in lung tissue (3). This was attributed to early increases in HAS 1 and HAS 2 mRNA expression and a decrease in Hyal 1 and Hyal 2 expression in the lung tissue (3). HA deposits provided sites for inflammatory cells to accumulate and supported subsequent collagen deposition (3). TSG-6 promoted HA deposition, suggesting the formation of HA–HC complexes, and eosinophilic airway inflammation and airway hyper-responsiveness (45), correlating HA deposits with the eosinophilic response. Further evidence for a role of HA in allergic airway inflammation comes from the use of a HA synthesis inhibitor, 4-methylumbelliferone (88), which reduces Ova-induced eosinophil airway inflammation (124). Thus, these HA deposits may support inflammation by sequestering inflammatory cells and providing survival or other signals. Alternatively, it is possible that the presence of HA deposits may reflect an attempt to resolve the inflammation by promoting matrix deposition. More work is needed to fully understand the role of HA deposits in the inflammatory response.

### A role for CD44 in resolving inflammation

#### CD44 facilitates the clearance of HA fragments and helps resolve sterile inflammation in the lung

The mouse bleomycin model of sterile inflammation and fibrosis has been used extensively by Noble and colleagues, and has provided key data on the role of HA and CD44 in the inflammatory process [reviewed in Ref. (97, 112)]. Bleomycin induces lung injury and necrosis that triggers an inflammatory response and subsequent wound repair mechanisms. Inflammatory monocytes and neutrophils are recruited to the damaged site, where they are activated by inflammatory signals released from the injured and necrotic tissue. These activated cells will phagocytose cell debris but will also produce anti-microbial factors such as ROS, which cause further tissue damage. HA is normally undetectable in the BALF but increases upon inflammation to approximately 2 μg/ml at the peak of the response. HA levels are also increased in the lung tissue upon inflammation, from 100 to 300 ng/mg of dry tissue. This increase is also accompanied by a decrease in molecular mass (500 kDa compared to the normal size of 1400 kDa) (19). Normally, the inflammatory response transitions to a healing response, where further neutrophil recruitment is halted, and debris and apoptotic neutrophils are scavenged by macrophages and/or inflammatory monocytes. Increased levels of TGFβ stimulate fibroblast proliferation and ECM production, which restores the tissue back to its original state. In CD44-deficient mice, the initial inflammatory phase appears normal, except for a higher accumulation of HA fragments. However, the CD44-deficient mice cannot resolve the inflammation: TGFβ activation is defective; apoptotic neutrophils are not cleared; and HA levels continue to rise in both the BALF and lung tissue (19). This points to a defect in clearance by macrophages or monocytes and a role for CD44 in this process. This defect is largely corrected by reconstitution of CD44-deficient mice with a CD44-sufficient immune system (19), further implicating CD44 on immune cells in the clearance of HA. The CD44-mediated uptake of HA and associated debris, together with CD44-assisted activation of TGFβ (125), may be sufficient to tip the balance toward the resolution phase of the inflammatory response. While a protective role for CD44 has also been observed in severe hypoxia-induced lung damage by promoting HA clearance and protecting from epithelial cell death (126), this role for CD44 in the resolution phase is not universally apparent, particularly when infections occur. This highlights potential differences between pathogenic and sterile inflammation and/or suggests that additional factors drive resolution when pathogens are encountered.

## Summary

The key points to emerge from this review are:

1. Few immune cells bind HA during homeostasis (alveolar macrophages are a notable exception).
2. The form of HA (its size, association with other molecules, and its ability to form complexes) changes between homeostasis and inflammation.
3. Immune cell interactions with HA increase upon an inflammatory response. This may help recruit immune cells to the site of inflammation as well as keep cells at the site and may facilitate their survival and function.
4. CD44 is the only receptor that has been demonstrated to bind HA on immune cells. HA binding by CD44 can lead to HA uptake and its subsequent degradation. Macrophage CD44 is thought to play an important role in HA uptake and the clearance of HA fragments during lung inflammation.
5. Additional evidence is required to establish whether purified HA fragments and HA oligomers are proinflammatory and if so, what receptors do they interact with to mediate this effect.
6. There is a need to better understand the composition and structure of the HA fragments and complexes present *in vivo* during an inflammatory response, and to then mimic their effects *in vitro*.

## Conflict of Interest Statement

The authors declare that the research was conducted in the absence of any commercial or financial relationships that could be construed as a potential conflict of interest.
